# Genomic and Transcriptomic Changes That Mediate Increased Platinum Resistance in *Cupriavidus metallidurans*

**DOI:** 10.3390/genes10010063

**Published:** 2019-01-18

**Authors:** Md Muntasir Ali, Ann Provoost, Laurens Maertens, Natalie Leys, Pieter Monsieurs, Daniel Charlier, Rob Van Houdt

**Affiliations:** 1 Microbiology Unit, Belgian Nuclear Research Centre (SCK•CEN), 2400 Mol, Belgium; md.muntasir.ali@sckcen.be (M.M.A.); ann.provoost@sckcen.be (A.P.); laurens.maertens@sckcen.be (L.M.); natalie.leys@sckcen.be (N.L.); pieter.monsieurs@sckcen.be (P.M.); 2 Research Group of Microbiology, Department of Bioengineering Sciences, Vrije Universiteit Brussel, 1050 Brussel, Belgium; dcharlie@vub.be; 3 Research Unit in Biology of Microorganisms (URBM), Faculty of Sciences, UNamur, 5000 Namur, Belgium

**Keywords:** platinum resistance, RNA-Seq, multireplicon, Nanopore, adaptive laboratory evolution

## Abstract

The extensive anthropogenic use of platinum, a rare element found in low natural abundance in the Earth’s continental crust and one of the critical raw materials in the EU innovation partnership framework, has resulted in increased concentrations in surface environments. To minimize its spread and increase its recovery from the environment, biological recovery via different microbial systems is explored. In contrast, studies focusing on the effects of prolonged exposure to Pt are limited. In this study, we used the metal-resistant *Cupriavidus metallidurans* NA4 strain to explore the adaptation of environmental bacteria to platinum exposure. We used a combined Nanopore–Illumina sequencing approach to fully resolve all six replicons of the *C. metallidurans* NA4 genome, and compared them with the *C. metallidurans* CH34 genome, revealing an important role in metal resistance for its chromid rather than its megaplasmids. In addition, we identified the genomic and transcriptomic changes in a laboratory-evolved strain, displaying resistance to 160 µM Pt^4+^. The latter carried 20 mutations, including a large 69.9 kb deletion in its plasmid pNA4_D (89.6 kb in size), and 226 differentially-expressed genes compared to its parental strain. Many membrane-related processes were affected, including up-regulation of cytochrome c and a lytic transglycosylase, down-regulation of flagellar and pili-related genes, and loss of the pNA4_D conjugative machinery, pointing towards a significant role in the adaptation to platinum.

## 1. Introduction

Platinum (Pt) is a rare element that is found in low natural abundance (0.4 parts per billion) in the Earth’s continental crust [1,2]. It is extensively used in industry, vehicle exhaust catalysts (VECs), and anticancer drugs [3], with cisplatin being one of the potent anti-cancer drugs in use [4]. Anthropogenic uses and emissions of platinum have resulted in increased concentrations (0.5–1.4 ton year^−1^) in surface environments, which could negatively impact natural habitats, especially because of the solubility of some forms of platinum [3,5]. It can enter waters, sediments, and soils and eventually reach the food chain [3]. Therefore, effective measures must be taken to minimize its spread and increase its recovery from the environment.

Platinum is also one of the critical raw materials in the EU innovation partnership framework, which were selected because of their high economic importance and high supply risk [6]. This makes platinum an important candidate for biological recovery from waste streams and other environmental niches. As bacterial communities have been naturally associated with platinum-group mineral grains [7], efforts have been made to explore the usability of microorganisms for the recovery of platinum group metals from the environment [8]. For instance, biological recovery of platinum has been shown for halophilic microbial communities, indicating that Pt from waste streams can be transformed into Pt-rich biomass, which in turn can be used as input for the refinery of precious metals [9]. In addition, Pt biosorption has also been studied for axenic bacterial cultures, including sulfate-reducing bacteria such as *Desulfovibrio desulfuricans, Desulfovibrio fructosivorans,* and *Desulfovibrio vulgaris* [10], as well as *Shewanella oneidensis*, *Cupriavidus metallidurans*, *Geobacter metallireducens*, *Pseudomonas stutzeri,* and *Bacillus toyonensis* [11,12]. The sulfate-reducing *Desulfovibrio* spp. and metal-ion reducing *Shewanella algae* have the capability to reduce Pt to zero state and form Pt nanoparticles in their periplasmic space [13,14]. It has been hypothesized that *Desulfovibrio* spp. can use Pt, as well as palladium, as terminal electron acceptors in their energy production pathway via cytochrome *c3* [15,16,17]. This promotes nanoparticle formation on the cell surface, preventing re-entry and acting as catalysts for further metal reduction [17]. *Cupriavidus* sp. also showed similar nanoparticle formation in the presence of palladium, according to an equivalent strategy [18].

Studying the interaction between platinum and bacteria showed that platinum inhibits cell division and enhances filamentous growth of *Escherichia coli* [19], *Caulobacter crescentus* and *Hyphomicrobium* sp. [20]. It inhibits DNA synthesis and DNA repair functions were shown to be essential for growth in the presence of platinum, as *E. coli* mutants deficient in DNA repair functions are unable to grow in the presence of platinum [21,22]. Similar to other DNA synthesis inhibitors, Pt also induces prophages from lysogenic *E. coli* strains [23]. The mutagenic ability of different platinum compounds has also been demonstrated in *Salmonella enterica* subsp. *enterica* serovar Typhimurium strains [24].

It is clear that most studies have analyzed the biological immobilization of Pt and its possible applications. However, only a limited number of studies focused on the effect of prolonged exposure to Pt, as would be the case for environmental bacteria in Pt-contaminated waters, soils, and sediments. For instance, Maboeta et al. showed that enzymatic activities and viable biomass were impacted in a platinum tailing disposal facility associated with mining activities [25].

In this study, we used *C. metallidurans* NA4 as a model to explore the adaptation of environmental bacteria to platinum exposure. It has been extensively studied for its resistance to a variety of metal (oxyan)ions [26,27,28]. We used a combinatorial sequencing approach to fully resolve the *C. metallidurans* NA4 genome consisting of six replicons [29], compared its genome with that of *C. metallidurans* CH34, and identified the genomic and transcriptomic changes in a laboratory-evolved strain, displaying increased resistance to platinum.

## 2. Materials and Methods

### 2.1. Strains, Media, and Culture Conditions

*C. metallidurans* NA4 was routinely cultured at 30 °C in Lysogeny broth (LB) or Tris-buffered mineral medium (MM284) supplemented with 0.2% (*w/v*) gluconate [30]. For culturing on solid medium, 1.5% agar (Thermo Scientific, Oxoid, Hampshire, UK) was added; liquid cultures were grown in the dark on a rotary shaker at 150 rpm. Metal salts used included PtCl_4_, Na_2_PdCl_4_, ZnSO_4_.7H_2_O, NiCl_2_.6H_2_O, CuSO_4_.5H_2_O and AgNO_3_. (Sigma-Aldrich, Overijse, Belgium).

### 2.2. Determination of the Minimal Inhibitory Concentration and Generation of Pt-Resistant Mutants

The minimal inhibitory concentration (MIC) of Pt^4+^, Pd^2+^, Zn^2+^, Ni^2+^, Cu^2+^, and Ag^+^ was determined using the broth dilution method in a 96-well plate containing a concentration gradient of the corresponding metals [31]. To select for *C. metallidurans* mutants displaying increased platinum resistance, a serial passage experiment was performed by continuous exposure to subinhibitory concentrations of Pt^4+^ using the gradient MIC method [32].

### 2.3. Plasmid Isolation and Restriction Digestion

The extraction of megaplasmids was based on the method proposed by Andrup et al. [33]. Extracted plasmid DNA was separated by horizontal gel electrophoresis (23 cm-long 0.5% Certified Megabase agarose gel (Bio-Rad, Temse, Belgium) in 1X Tris-Borate-EDTA buffer, 100 V, 20 h) in a precooled (4 °C) electrophoresis chamber. After GelRed staining (30 min + overnight destaining at 4 °C in ultrapure water), DNA was visualized and images captured under UV light transillumination (Fusion Fx, Vilber Lourmat, Collégien, France). To confirm the presence and size of the smaller plasmid in NA4Pt (Pt^4+^ resistant mutant of NA4), plasmid DNA was isolated with the Wizard^®^ Plus SV Miniprep DNA Purification System (Promega, Leiden, The Netherlands). The isolated DNA was used for restriction digestion with *Pag*I (Fisher Scientific, Merelbeke, Belgium). The products were separated on a 0.6% agarose (Molecular Biology Grade, Eurogentec, Belgium) gel to visualize the individual fragments together with the GeneRuler 1 kb plus ladder (Fisher Scientific, Merelbeke, Belgium).

### 2.4. Motility, Scanning Electron Microscopy (SEM), and Flow Cytometry

For testing motility, *C. metallidurans* NA4 and NA4Pt were grown in LB media until the OD_600_ reached 0.6. Five µL of the culture was then stab inoculated onto a LB plate containing 0.3% agar. The radius of the growth pattern was measured after 24 h.

For SEM, cells were grown in MM284 in normal growth conditions, centrifuged (5000 rpm for 8 min), washed in Milli-Q water, and fixed with 3% glutaraldehyde solution at 4 °C (3 h). Cells were sputter coated (22 nm) with gold and examined under SEM at an accelerating voltage of 10 kV.

For flow cytometry, bacterial cell suspensions (OD_600_ = 0.6) were diluted 1000 times in 0.2 μm filtered Tris-buffered mineral medium (MM284), Next, SYBR green (Sigma Aldrich) dye was added and incubated at 37 °C for 20 min. Stained bacterial suspensions were analyzed on the Accuri C6 flow cytometer (BD, Erembodegem, Belgium).

### 2.5. Genome Sequencing

Total DNA from *C. metallidurans* NA4 and NA4Pt was isolated using the QIAamp DNA mini kit (Qiagen, Venlo, the Netherlands). The parental strain NA4 [29] was resequenced using a combination of Illumina and Nanopore sequencing. Illumina sequencing was performed on the Illumina HiSeq 2500 platform using 2 × 75 bp paired-end sequencing (Baseclear, Leiden, Netherlands). Nanopore sequencing was performed in-house using the MinION device with an R9 flow cell and the Rapid Sequencing kit. Strain NA4Pt was sequenced using the Illumina Miseq platform (40× coverage; MicrobesNG, Birmingham, UK).

### 2.6. Genome Assembly

Genome assembly was performed using the pre-assembled contigs based on the 454 sequencing data as “trusted contigs” combined with the illumina and nanopore sequencing data as input for the SPAdes algorithm (version 3.11.1, default parameter settings) [34,35]. Subsequent genome polishing was performed by consecutive runs of an in-house Perl script, where the original reads were realigned against the resulting assembly using the Burrows-Wheeler Aligner (BWA). Based on this output, the genome assembly was updated accordingly until no further single-nucleotide polymorphisms (SNPs) and indels were detected.

The *C. metallidurans* NA4Pt genome was compared to the parental strain at two levels. For the small SNPs and indels, the output of two algorithms was combined: BreSeq 0.32.0 and the Genome Analysis Toolkit (GATK) [36,37]. BreSeq was run using the default parameter settings. Before running GATK, we first converted the raw BWA output to a sorted and index Binary Alignment Map (BAM) file using the view, sort and index command of the SAMtools package version 0.1.18 [38]. SNP prediction was performed on this BAM file by following the pipeline described in Van der Auwera et al. [37], with default parameters, and using ploidy = 1 when running the HaplotyperCaller command. For larger structural variations (insertions and deletion), an in-house developed Python script, specifically focused on the identification of structural variations caused by mobile genetic elements, was used. This program exploits the paired-end information and insert size distributions to predict these variations.

### 2.7. Transcriptomic Analysis Using RNA-Seq

Gene expression in NA4Pt was compared with the parental strain NA4 under non-selective growth conditions. Three independent *C. metallidurans* NA4 and NA4Pt cultures were allowed to grow until an OD_600_ of 0.6 was reached. Each culture was subdivided in 2 mL portions and cells were harvested by centrifugation for 2 min at 10,000× *g*. Bacterial pellets were flash frozen by immersion into liquid nitrogen and kept frozen at −80 °C at all times. Total RNA was extracted using the Promega SV Total RNA Isolation System kit (Promega, Leiden, The Netherlands). RNA sequencing (directional mRNA library, RiboZero rRNA depletion and 2 × 125 bp paired-end sequencing) was performed by Eurofins genomics (Ebersberg, Germany).

### 2.8. RNA-Seq Data Analysis

Obtained RNA-Seq reads were aligned using BWA software and the default parameters [39]. Raw counts per gene were calculated based on the latest genome annotation of *C. metallidurans* NA4, as available on the MaGe platform. Reads were allowed to map 50 bp upstream of the start codon or 50 bp downstream of the stop codon. Reads that were mapped to ribosomal or transfer RNA were removed from the raw count data to prevent bias in detecting differential expression. Differential expression was calculated using the edgeR package (version 3.2.4) [40] in BioConductor (release 3.0, R version 3.1.2), resulting in a fold change value and a corresponding *p* value corrected for multiple testing for each gene. Genes were found to be differentially expressed if they show an absolute log2 fold change higher than 0.80 and a false discovery rate (FDR) value lower than 0.05.

### 2.9. Functional Analysis

Homologous genes and synteny groups were computed via the MaGe platform [41]. Homologous genes were based on the bidirectional best hit criterion and a blastP alignment threshold (at least 35% amino-acid identity on 80% of the length of the smallest protein). Synteny, orthologous gene sets that have the same local organization are based on the bidirectional best hit criterion or a blastP alignment threshold (at least 30% amino-acid identity on 80% of the length of the smallest protein), and co-localization (with the maximum number of consecutive genes not involved in a synteny group being five).

Distribution of insertion sequence (IS) elements was determined by identification and annotation of IS elements with ISsaga [42] and manual curation.

MOB typing of plasmids was performed using the mob_typer script, part of the MOB-suite [43]. All analyses were performed using standard parameters. The most recent reference database was downloaded on the 19th of November, 2018.

Plasmids were aligned to pNA4_D by the AliTV perl interface in the AliTV package [44] using standard parameters. Output json files were visualized with the AliTV web service (http://alitvteam.github.io/AliTV/d3/AliTV.html). Subsystem categories were assigned via the online implementation of RASTtk (Rapid Annotation using Subsystem Technology) (http://rast.nmpdr.org/) [45]. Circos plots from genomic data were constructed with Circa 1.2.1 (http://omgenomics.com/circa/).

## 3. Results and Discussion

### 3.1. Genome Analysis of C. metallidurans NA4

The *C. metallidurans* NA4 genome was previously sequenced via the 454 GS-FLX sequencing platform [29] and assembled into 109 contigs. Since all *Cupriavidus* genomes have a multipartite organization composed of at least a chromosome, a chromid and one or more megaplasmids [27], we tested if combining sequencing platforms could produce completely closed replicon sequences. *C. metallidurans* NA4, in particular, contains six replicons [29,46]. Complete assembly of multipartite genomes is necessary to fully understand the genomic structure and invaluable to correctly assess genomic events. An additional Illumina and Nanopore sequencing was performed. Integration of the pre-assembled contigs (based on the 454 pyrosquencing data) and the Illumina and Nanopore sequencing data, using the SPAdes platform, resulted in complete assembly and closure of the six *C. metallidurans* NA4 replicons (Table 1). The long reads from Nanopore sequencing resolved the complex repeat regions, while Illumina sequencing provided more accurate sequencing results.

**Figure 1:**
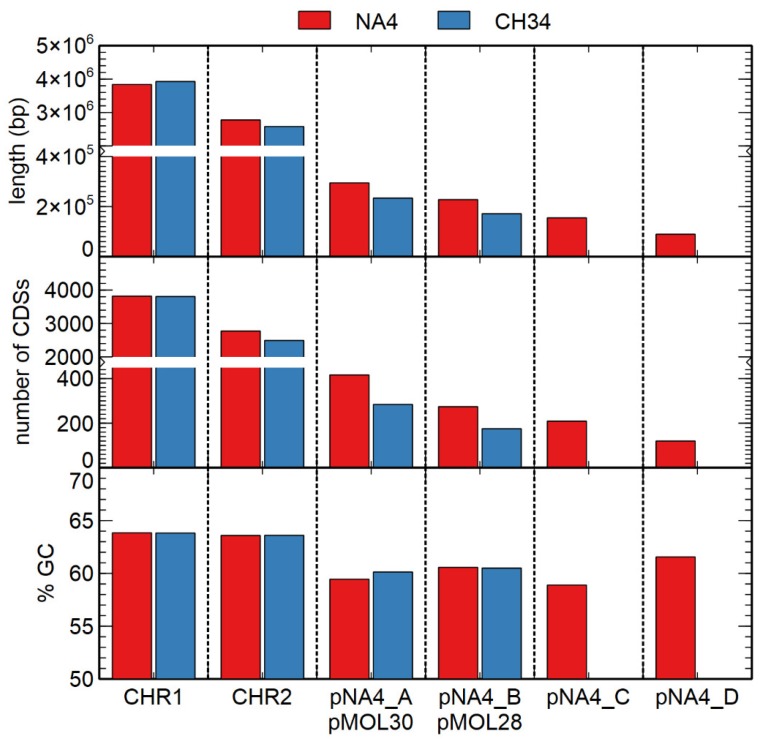
Comparison of the size (bp), number of coding sequences (CDSs), and GC content (% GC) of the *C. metallidurans* NA4 (red) and CH34 (blue) genome replicons (CHR1: chromosome; CHR2: chromid; p: plasmids).

**Figure 2:**
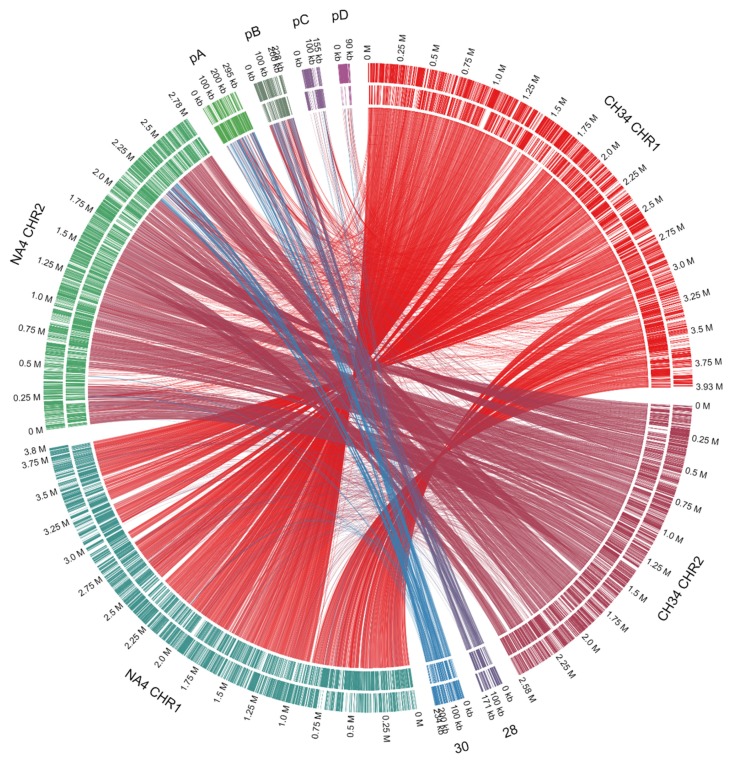
Circa plot of the *C. metallidurans* CH34 and NA4 genomes (CHR1: chromosome; CHR2: chromid) and plasmids (28: pMOL28; 30: pMOL30; pA: pNA4_A; pB: pNA4_B; pC: pNA4_C; pD: pNA4_D). Connections correspond to homologous genes based on the bidirectional best hit criterion and a blastP alignment threshold (at least 35% amino-acid identity on 80% of the length of the smallest protein).

**Figure 3:**
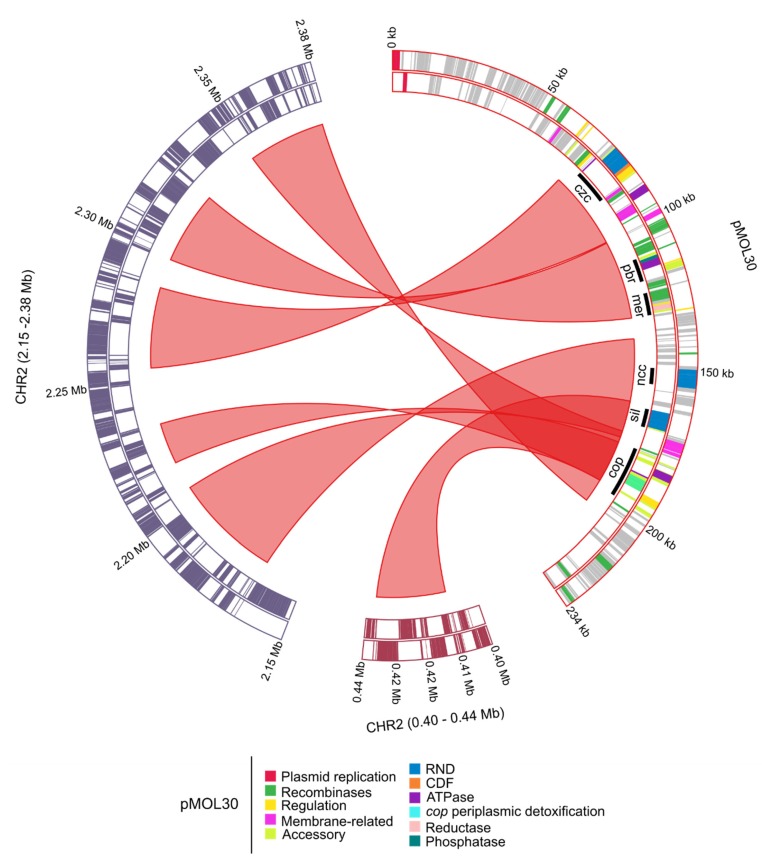
Circa plot of pMOL30 from *C. metallidurans* CH34 and sections of the *C. metallidurans* NA4 chromid (CHR2). Ribbons correspond to synteny groups, orthologous gene sets that have the same local organization, based on the bidirectional best hit criterion or a blastP alignment threshold (at least 30% amino-acid identity on 80% of the length of the smallest protein) and co-localization (with the maximum number of consecutive genes not involved in a synteny group being five). The pMOL30 genes are colored according to their function.

**Figure 4:**
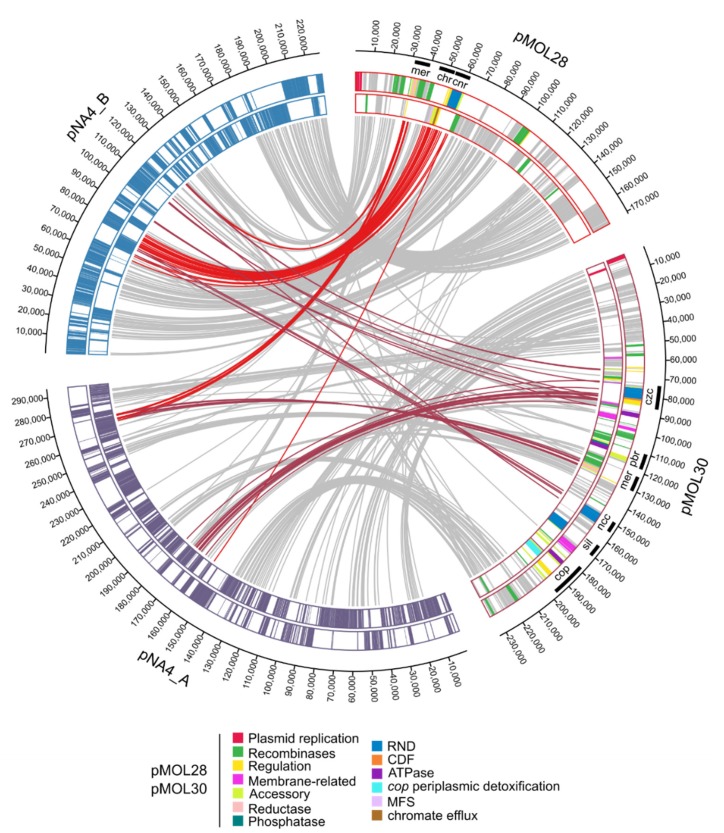
Circa plot of pMOL30 and pMOL28 from *C. metallidurans* CH34 and pNA4_A and pNA4_B from *C. metallidurans* NA4. Connections correspond to homologous genes based on the bidirectional best hit criterion and a blastP alignment threshold (at least 35% amino-acid identity on 80% of the length of the smallest protein). Colored connections correspond to genes involved in metal resistance. The pMOL28 and pMOL30 genes are colored according to their function.

The size, number of coding sequences (CDSs) and GC content of the *C. metallidurans* NA4 chromosome and chromid were very similar to that of type strain *C. metallidurans* CH34 [26] (Figure 1). To compare the different replicons of CH34 and NA4, homologous genes were computed and visualized by Circa (Figure 2). Both the chromosome (CHR1) and chromid (CHR2) were well conserved. However, this comparison revealed an extensive number of homologous genes between plasmid pMOL30 from CH34 and the chromid of NA4 (Figure 2). To zoom in on this, large (>15 genes) synteny groups between pMOL30 and the chromid of NA4 were visualized separately (Figure 3), as well as homologous genes shared between pMOL28 and pMOL30, and pNA4_A and pNA4_B (Figure 4).

The latter indicated that the main large metal resistance clusters on pMOL30 are syntenic with gene clusters on NA4’s chromid instead of NA4’s plasmids. Previously, we showed that pMOL28 and pMOL30 contain large genomic islands that harbor all plasmid-borne genes involved in the response to heavy metals [27,47,48]. Plasmid pMOL30 carries two genomic islands, CMGI-30a and -30b, that convey resistance to cadmium, zinc, cobalt, lead, and mercury, and copper and silver, respectively. A 25-gene cluster within CMGI-30a, containing the *czc* cluster (related to cadmium, zinc and cobalt resistance) and genes involved in membrane-related functions, and highly conserved (>99% nucleotide similarity) in all *C. metallidurans* strains [48], was found on NA4’s chromid. Interestingly, this cluster is always flanked by a tyrosine-based site-specific recombinase (TBSSR) associated with a conserved protein of unknown function making up a bipartite module (BIM) [48,49]. Our observation adds evidence to the mobility of this cluster. The cluster on NA4’s chromid related to the *pbr* (lead resistance) and *mer* (mercury resistance) cluster (Tn*4380*) of pMOL30 was flanked at both sides by (remnants of) TBSSRs (only at one side for pMOL30). Three clusters on NA4’s chromid were homologous to pMOL30’s *cop* cluster (copper resistance) (Figure 3, Supplementary Figure S1). The latter was part of genomic island CMGI-30b of pMOL30 that contains a 33-gene copper-related cluster almost completely induced by Cu^2+^ (coding for the efflux P_I_-type ATPase CopF, the heavy metal efflux (HME) resistance nodulation cell-division (RND) system SilCBA, the periplasmic detoxification system CopABCDI, and accessory and membrane-related functions) as well as the *nre*/*ncc* cluster. One NA4 cluster comprised almost the complete CMGI30-b island except *copV* and *copT*. A second cluster contained the copper-related cluster as well as some gene fragments and was flanked by TBSSRs. The third cluster comprised the *cop* cluster except *copV* and *copT* (Supplementary Figure S1). In addition, a cluster carrying resistance to chromate and cobalt/nickel, similar to that on genomic island CMGI-28a of pMOL28, was also found on NA4’s chromid (next to plasmid pNA4_B). The cluster was delimited by IS*Rme1* and a TBBSR, and was located between the *pbr* and *mer* cluster, and the second *cop* cluster. It did not contain the additional five genes that are carried by the pMOL28 and pNA4_B cluster [27,50,51].

Although most metal resistance determinants are conserved between *C. metallidurans* NA4 and CH34, which results in similar growth profiles in the presence of metals [52], they are harbored by different replicons. We showed that, unlike the characteristic megaplasmid pMOL30 in *C. metallidurans* CH34 that is specialized in heavy metal resistance, pNA4_A does not fulfill this role in *C. metallidurans* NA4. Plasmid pNA4_B does harbor the pMOL28-like metal resistance determinants and, in addition, a second *nccYXHCBAN* locus coding for an RND-driven efflux system homologous to that of *C. metallidurans* 31A and KT02, which mediates resistance to 40 mM Ni^2+^.

#### 3.1.1. Characterization of (Mega)Plasmids

As our sequencing efforts resulted in the complete closure of the four plasmid replicons, we were able to characterize them in more detail. Numerous proteins are involved in the horizontal transmission of plasmids via conjugation and establishment in the recipient cell. Conjugative plasmids contain an origin of transfer (*oriT*), a DNA relaxase, a Type IV coupling protein (T4CP), and a membrane-associated mating pair formation (MPF) complex, which is a form of Type IV secretion system (T4SS). Transmissible (mobilizable) plasmids require an *oriT* and a relaxase that can be provided in trans [53,54,55,56,57]. Different classification or typing schemes for plasmids have been developed, but the principal ones are replicon and MOB typing, relying on plasmid replication and mobility, respectively [55,56,58]. The phylogenetic relationship among relaxases has been thoroughly studied and resulted in the classification of conjugative systems into six MOB families: MOB_F_, MOB_H_, MOB_Q_, MOB_C_, MOB_P_, and MOB_V_ [43,56]. The archetype plasmids defining the families are R388 (MOB_F_), R27 (MOB_H_), RP4 (MOB_P_), RSF1010 (MOB_Q_), pMV158 (MOB_V_), and CloDF13 (MOB_C_). We used MOB-suite to type and characterize pNA4_A, pNA4_B, pNA4_C, and pNA4_D, as well as pMOL28 and pMOL30 (Table 2).

Our analysis indicated that pNA4_A and pMOL30, and pNA4_B and pMOL28 were similar based on replicon, relaxase and mating pair formation (MPF) family. Plasmid pNA4_A was classified as being non-mobilizable, whereas pMOL30 as conjugative. Although low-frequency transfer of pMOL30 has been observed, this transfer could be mediated via other conjugative systems. For instance, plasmid RP4 can enhance transfer frequency to 10^−3^ by cointegrate formation via transposition of Tn*4380* [47]. Plasmid pNA4_A carried 416 CDSs, most of them code for unknown proteins (71.6%). Identifiable functions were, next to plasmid replication, maintenance and conjugation, related to metal resistance (partial *czc* cluster and mercury transposon; see Figure 4) and alkaline phosphatase. Plasmid pNA4_B carried 274 CDSs, most of them also code for unknown proteins (59.5%). Identifiable functions were, next to plasmid replication, maintenance and conjugation, related to metal resistance (cluster carrying resistance to chromate and cobalt/nickel, similar to that on genomic island CMGI-28a of pMOL28; see Figure 4).

Plasmid pNA4_C was classified as non-mobilizable and carried 209 CDSs, most of them also code for unknown proteins (74.6%). No accessory plasmid functions could be identified. Plasmid pNA4_D was classified as conjugative and was very similar to plasmids from *Acidovorax carolinensis* P3 (plasmid pACP3.3) and P4 (plasmid pACP4.4), *Acidovorax* sp. JS42 (plasmid pAOVO01), *Alicycliphilus denitrificans* K601 (plasmid pALIDE201), and *Pandoraea pnomenusa* MCB032 (unnamed plasmid) (Supplementary Figure S2). Characterized proteins encoded by these plasmids were mainly related to conjugational transfer and replication.

#### 3.1.2. Insertion Sequence Elements Distribution

Insertion sequences (IS) are simple mobile genetic elements that play an important role in genome plasticity and activity of these IS elements are often correlated with the adaptive potential to promote genetic variability under different environmental challenges [59,60]. An initial assessment of the number and identity of IS elements in NA4 was previously performed based on the draft genome assembly [52]. However, this could lead to an underestimation of the number of IS elements because possible identical IS elements will only be represented as one contig [61]. Therefore, we reanalyzed and determined the correct number of IS elements in NA4 (Table 3). In total, 21 intact IS elements were identified. *C. metallidurans* NA4 carried much less IS elements in comparison with type strain *C. metallidurans* CH34, which carried 57 intact IS elements.

### 3.2. Analysis of C. metallidurans NA4Pt

#### 3.2.1. Determination of Minimal Inhibitory Concentration and Generation of a Pt^4+^ Resistant Mutant

**Figure 5:**
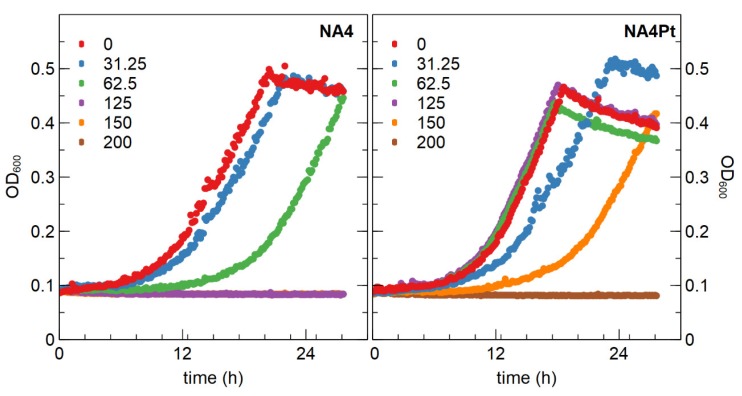
Growth of *C. metallidurans* NA4 and NA4Pt in the presence of different PtCl_4_ concentrations (in µM).

We used *C. metallidurans* NA4, which is able to survive in oligotrophic conditions for many months [46], to scrutinize adaptation to Pt^4+^. The minimal inhibitory concentration (MIC) of Pt^4+^ for *C. metallidurans* NA4 in Tris-buffered mineral medium was 70 µM (Table 1), which was similar to that of *C. metallidurans* CH34. This already indicated that NA4 has a high level of resistance to Pt^4+^ when compared to other strains such as *Klebsiella pneumoniae* (20 µM), *Acinetobacter baumannii* (30 µM), and *Enterococcus faecium* (60 µM) [63]. Furthermore, the MIC determinations in that study were performed in rich broth medium, which could affect the metal bioavailability and lead to an overestimation of the MIC [63]. Next, *C. metallidurans* NA4 was exposed to a subinhibitory concentration of 62.5 µM Pt^4+^ during 30 days (eight serial passages). After passage on non-selective medium, a mutant (designated NA4Pt) that displayed a higher resistance to Pt^4+^ (MIC of 160 µM) was obtained. No differences were observed between NA4 and NA4Pt when grown in non-selective conditions (Figure 5). In addition, no differences in MIC of Pd^2+^, Ag^+^, Zn^2+^, Ni^2+^, and Cu^2+^ were observed (Table 4). From this data it is evident that the NA4Pt mutant is specifically resistant to Pt^4+^ and does not have a higher resistance to Pd^2+^, in contrast to what was described for *Desulfovibrio* sp. preference during biosorption [14].

#### 3.2.2. Sequence Analysis of *C. metallidurans* NA4Pt

**Figure 6:**
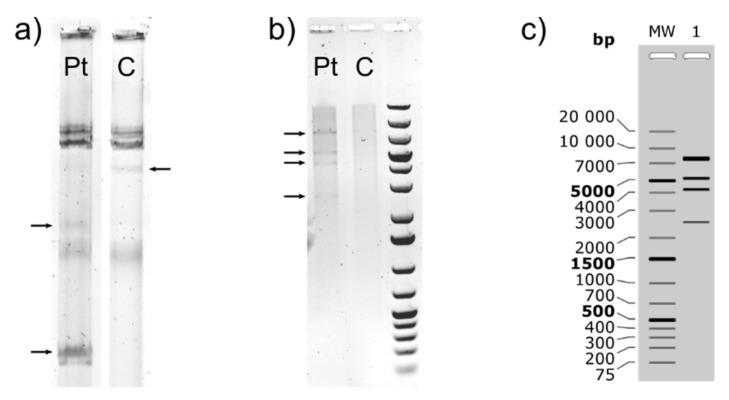
Agarose gel electrophoresis of *C. metallidurans* NA4 (C) and NA4Pt (Pt) plasmid DNA. (**a**) Megaplasmids of NA4 vs. NA4Pt; (**b**) *Pag*I digest of pNA4_D in NA4 vs. NA4Pt (only small plasmids were extracted; therefore, no discrete bands are visible for NA4); and (**c**) theoretical *Pag*I digest of NA4Pt pNA4_D.

The laboratory-evolved mutant NA4Pt was sequenced to identify genomic changes such as insertions, deletions and SNPs (Supplementary Table S1). These genomic changes were not observed in other adaptive laboratory evolution experiments with NA4 (unpublished results). In the chromosome, an insertion (+56 bp) in the upstream region of a metal-dependent hydrolase and a point mutation in pseudouridine synthase (*rluB*) (resulting in R64H substitution) were observed. In the chromid, several point mutations (mostly synonymous mutations) and two deletions (a single bp and a 119-bp region), both located in the *copB* gene coding for an outer membrane protein involved in copper resistance, were found. The latter did not affect copper resistance of NA4Pt (Table 4). The biggest change, a large deletion of 69.9 kbp, was observed in plasmid pNA4_D. The 19.7 kbp remaining fragment (positions 52,551 to 72,345) included genes encoding a DNA primase, a C-5 cytosine-specific DNA methylase, a single-stranded DNA-binding (ssb) protein, and the replication initiator protein RepA (Supplementary Table S2), which is responsible for plasmid replication in bacteria [64,65]. This suggested that the remaining part could be maintained as a smaller plasmid instead of being integrated in one of the other replicons. The absence of the native pNA4_D and presence of the smaller plasmid were confirmed by plasmid DNA extraction and *Pag*I digestion (Figure 6).

The large deletion, which could probably be mediated by the presence of a 221 bp direct repeat (Supplementary Figure S3), resulted in the loss of 89 CDSs. (Mega)plasmids are nonessential and dispensable for cell viability in most environments [66], and large deletions have also been observed in other laboratory-evolved strains. For instance, a large deletion occurred in the megaplasmid pAtC58 from *Agrobacterium tumefaciens* laboratory-evolved strains, resulting in increased virulence gene expression and reduced fitness cost [67]. Prolonged cultivation of the Gram-positive actinobacterium *Rhodococcus opacus* 1CP containing megaplasmid p1CP (740 kb) under non-selective conditions led to the isolation of mutants 1CP.01 and 1CP.02, harboring the shortened plasmid variants p1CP.01 (500 kb) and p1CP.02 (400 kb) [68]. *Methylobacterium extorquens* AM1 lost 10% of its megaplasmid in an evolution experiment, which were beneficial in the applied selective environment, but disadvantageous in alternative environments [69]. Gene loss is a very common evolutionary process in bacteria and provides an increased fitness under one or several growth conditions [70]. The large deletion in pNA4_D resulted in loss of the conjugative machinery (T4SS), which is known to impose a burden [71,72]. For instance, the growth rate of an experimentally evolved *E. coli* increased by IS*26*-mediated loss of the T4SS on its plasmid pKP33 [73]. Several mechanisms are put forward to explain the reduced fitness costs, such as ribosome occupancy, reduction of energy demands for DNA replication, transcription and translation, and negative interactions between chromosomal pathways and (mega)plasmid-encoded proteins [66,67,74]. In addition, selective processes favoring adaptation to specific stressors/environments can be a driving force behind gene loss [66,70,75,76]. Therefore, although no growth differences were observed between NA4 and NA4Pt, this loss could have an effect in challenging environments with increased selection pressure (e.g., high platinum concentration).

#### 3.2.3. Transcriptome Analysis

**Figure 7:**
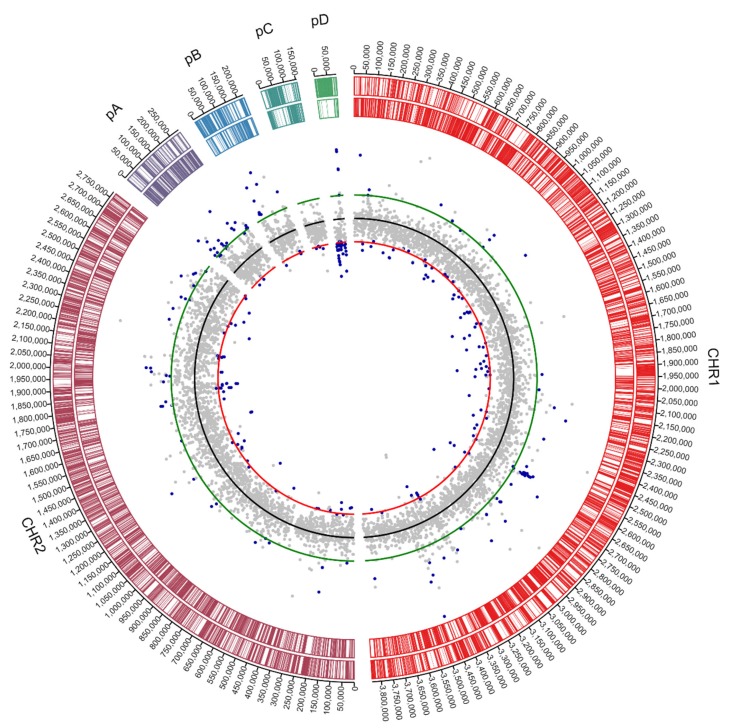
Scatter plot of RNA-Seq-derived gene expression of *C. metallidurans* NA4Pt compared to its parental strain in non-selective conditions. Dots (blue *p* < 0.05) represent Log2 ratios with red, black, and green lines corresponding to −0.8, 8, and 0.8, respectively.

Resistance is the result of natural selection for resistance-conferring mutations (i.e., random mutations that allow growth under selection, outcompeting the parent, and subsequent isolation of the adapted mutant) [77]. Therefore, and similar to other studies [78,79,80], the global shift in transcriptome, resulting from the altered genotype of the evolved strain (NA4Pt) as compared with the parental strain (NA4), was examined by RNA-Seq in non-selective conditions (average total number of reads was 4,324,281 ± 463,666). Up- and down-regulated genes were selected based on log2 fold change (<−0.8 and >0.8) and significance (*p* < 0.05), which resulted in 111 up- and 115 down-regulated genes (Figure 7 and Supplementary Tables S2 and S3).

**Figure 8:**
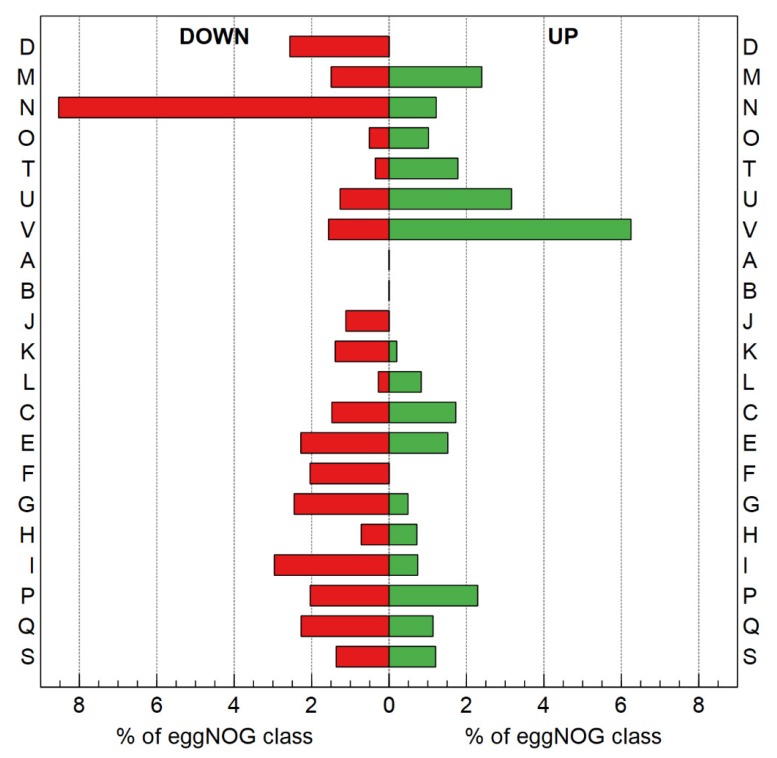
EggNOG classification of differentially expressed genes in *C. metallidurans* NA4Pt, based on RNA-Seq-derived gene expression of *C. metallidurans* NA4Pt compared to its parental strain in non-selective conditions. Percentages were calculated by normalizing the number of up- and down-regulated genes in each category by the total number of genes in the NA4 genome grouped in the corresponding category. (D: Cell cycle control, cell division, and chromosome partitioning; M: Cell wall/membrane/envelope biogenesis; N: Cell motility; O: Posttranslational modification, protein turnover, and chaperones; T: Signal transduction mechanisms; U: Intracellular trafficking, secretion, and vesicular transport; V: Defense mechanisms; A: RNA processing and modification; B: Chromatin structure and dynamics; J: Translation, ribosomal structure, and biogenesis; K: Transcription; L: Replication, recombination, and repair; C: Energy production and conversion; E: Amino acid transport and metabolism; F: Nucleotide transport and metabolism; G: Carbohydrate transport and metabolism; H: Coenzyme transport and metabolism; I: Lipid transport and metabolism; P: Inorganic ion transport and metabolism; Q: Secondary metabolites biosynthesis, transport, and catabolism; S: Poorly characterized).

The functional relevance of differentially-expressed genes was explored by using functional categories from the eggNOG classification system [81]. Genes of different categories were found to be differently expressed (Figure 8). However, none of the categories were significantly over-represented (Fisher’s exact test).

Overall, genes involved in defense mechanisms, intracellular trafficking, signal transduction and membrane-related genes were more up-regulated than down-regulated. On the other hand, genes involved in carbohydrate transport, nucleotide transport, cell motility and cell-cycle-related genes were more down-regulated than up-regulated. Up-regulated defense mechanism genes included mainly RND-driven efflux systems, which are abundant in the NA4 genome [52]. Next to systems putatively involved in the efflux of chemicals (acridines), the expression of two HME-RND genes was increased. The *cusC* gene is part of the CusCBA efflux pump responsible for copper and silver resistance [82]. In *C. metallidurans*, *cusDCBAF* genes are also up-regulated by silver ions [28], and induction of the CusC protein synthesis was also observed in the presence of 1 µM silver or 0.85 mM copper ions [83]. The *cnrC* gene is part of *cnrCBAT* efflux system that mediates nickel and cobalt resistance [84]. However, the resistance of NA4Pt to Cu^2+^, Zn^2+^, Ag^+^ and Ni^2+^ was unaffected compared to that of the parental strain NA4 (Table 4). No other metal resistance genes were up- or down-regulated.

Up-regulated genes of the signal transducing pathways belong to different two-component-system-related proteins (Supplementary Table S2). Namely, *ompR*-family regulators, members of the largest response regulator family involved in many signal transduction processes [85], and *cheY*, which is involved in chemotaxis and modulates motility by regulating flagellar motor switch proteins [86]. In contrast to *cheY*, cell-motility-related genes were down-regulated (*flgC*, *flgD*, *flgG*, *pilW,* and *pilX*). FlgC and FlgG proteins form the rod part of the flagellar basal body [87] and FlgG polymerizes to form the distal rod on top of the proximal rod, acting as a hook cap [88]. PilX and PilW are involved in biogenesis of Type IV fimbriae, which are surface filaments mediating attachment to host epithelial cells and flagella-independent twitching motility [89]. These differences did not affect motility as no significant differences were observed between NA4Pt and NA4 in motility assays (Supplementary Figure S4).

The class of membrane-related up-regulated genes contains a highly up-regulated (36.3-fold) lytic transglycosylase (LT), which usually plays an important role in shaping the periplasmic space and is tightly regulated, as over activity can have deleterious effects [90]. LT is responsible for creating space within the peptidoglycan layer for cell division and the insertion of cell-envelope spanning structures such as flagella and secretion systems [90]. In *C. metallidurans*, different LTs are induced in the presence of Cu^2+^, Cd^2+^, Pb^2+^ and Zn^2+^ [91]. Therefore, it may be hypothesized that the LTs might play a role in coping with toxic metal ion concentrations. Next to LTs, membrane-bound cytochrome c, which enables electron transfer as well as the catalysis of various redox reactions [92], is also up-regulated. In *D. vulgaris,* this periplasmic protein is directly involved in H_2_-mediated metal reduction [16]. *Desulfovibrio* sp. cytochrome c3 mediates electron transfer to palladium and platinum complexes, thereby reducing them to zero-valent nanoparticles [10,17]. The latter supports a role of this overexpressed gene in the Pt-resistant phenotype. Furthermore, nm-scale colloidal platinum was found in *C. metallidurans* primarily along the cell envelope, where energy generation/electron transport occurs [12].

**Figure 9:**
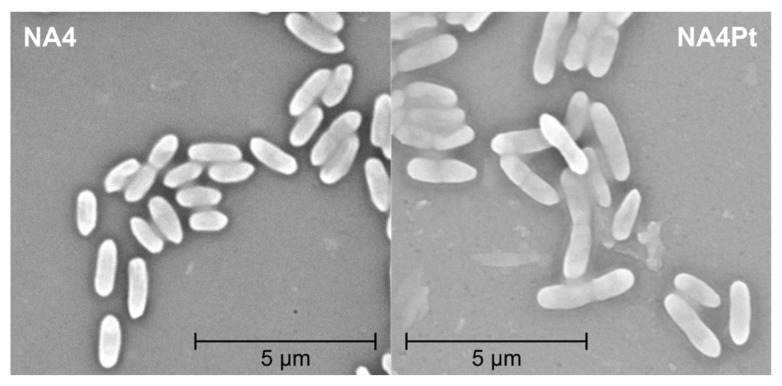
Scanning electron microscopy (SEM) image of *C. metallidurans* NA4 and NA4Pt.

To scrutinize the impact of differentially-expressed membrane-related genes (LT, flagellar- and pili-related) on morphology, scanning electron microscopy (SEM) was performed and showed that NA4Pt cells appeared to be more elongated (Figure 9). This correlates with the induction of filaments during platinum metal stress [19]. Nevertheless, the presence of a subpopulation of elongated cells could not be confirmed by flow cytometry (Supplementary Figure S5).

Up-regulated intracellular trafficking genes were mainly related to a Type II secretion system (T2SS), which secretes proteins to the extracellular environment [93]. In *C. metallidurans*, the T2SS secretes alkaline phosphatase in the extracellular environment [94].

Finally, none of the CDSs affected by the genomic changes in NA4Pt (either directly or via alteration in their promoter region) were differentially expressed (Supplementary Tables S1–S3). The latter points towards a significant role of the large deletion in pNA4_D on the overall expression pattern, as well as adaptation to platinum.

## 4. Conclusions

We resolved the multireplicon genome of *C. metallidurans* NA4 by combining Illumina and Nanopore sequencing and; thereby, paved the way for genetic, genomic and evolution-related studies. Further analysis of the replicons showed a distinctive pattern in the location of metal resistance determinants, with the chromid playing a pivotal role in contrast to type strain CH34, for which the megaplasmids pMOL28 and pMOL30 are the main actors. An NA4 derivative (NA4Pt) that showed increased resistance to platinum was generated via an adaptive laboratory evolution experiment. On the basis of our observations, one might speculate that the increased resistance to Pt^4+^ in *C. metallidurans* NA4Pt is not mediated by a dedicated mechanism, but by pleiotropic alterations in membrane-related processes, such as pili, peptidoglycan turnover and electron transfer, probably elicited by the large deletion in its 98-kb plasmid.

## Figures and Tables

**Table 1:** Overall characterization of the replicons constituting the *C. metallidurans* NA4 genome.

Replicon	Length (bp)	% GC ^1^	# CDSs ^2^
Chromosome	3,838,195	63.84	3818
Chromid	2,776,395	63.59	2774
Plasmid pNA4_A	294,575	59.45	416
Plasmid pNA4_B	227,796	60.57	274
Plasmid pNA4_C	155,041	58.90	209
Plasmid pNA4_D	89,606	61.56	120

^1^ Guanine-cytosine content; ^2^ Coding sequences.

**Table 2:** Plasmid characterization.

Replicon	Rep Type	Relaxase Type ^1^	MPF Type ^2^	Predicted Mobility	MASH Nearest Neighbor ^3^
pMOL28	591	MOB_H_	MPF_T_	Conjugative	-
pMOL30	332	(MOB_P_)	MPF_F_	Conjugative	-
pNA4_A	332	-	MPF_F_	Non-mobilizable	pMOL30
pNA4_B	591	MOB_H_	MPF_T_	Conjugative	pMOL28
pNA4_C	-	-	-	Non-mobilizable	pHS87a (*Pseudomonas aeruginosa*)
pNA4_D	1864	MOB_F_	MPF_F_	Conjugative	pACP3.3 (*Acidovorax* sp. P3)

^1^ Archetype relaxase: plasmid R388 (MOB_F_), plasmid R27 (MOB_H_), and plasmid RP4 (MOB_P_). ^2^ Archetype mating pair formation (MPF) complex: plasmid F (MPF_F_) and plasmid Ti (MPF_T_). ^3^ MASH Nearest Neighbor are not included for pMOL28 and pMOL30, as the closest database match was a self-hit.

**Table 3:** Distribution of insertion sequence elements in *C. metallidurans* NA4.

Element	Family	Size (%) ^1^	CHR1	CHR2	pNA4_A	pNA4_B	pNA4_C	pNA4_D
IS*1071*	Tn*3*	3204 (99.9%)						1
		2991 (93.4%)			2			
IS*Rme4*	IS*21*	2469 (100%)	4	3				
IS*Rme9*	IS*21*	2674 (94.8%)					1	
IS*Rme10*	IS*30*	1063 (100%)		1				
IS*Rme3*	IS*3*	1288 (100%)	1	2		2		
IS*Pst3*	IS*21*	2605 (97.8%)	1	2				
IS*Pa45*	IS*4*	1637 (100%)	1					

^1^ Size (bp) of the element and % nucleotide sequence similarity to the insertion sequence (IS) element as defined in ISFinder [62].

**Table 4:** Minimal inhibitory concentration of selected metals for *C. metallidurans* NA4 and NA4Pt.

	Pt^4+^ (µM)	Pd^2+^ (µM)	Ag^+^ (µM)	Zn^2+^ (mM)	Ni^2+^ (mM)	Cu^2+^ (mM)
NA4	70	12.5	1	12	40	6
NA4Pt	160	12.5	1	12	40	6

## Supplementary Materials

The following are available online at http://www.mdpi.com/2073-4425/10/1/63/s1, Figure S1: *C. metallidurans* chromid clusters homologous to the *cop* cluster on pMOL30; Figure S2: Whole plasmid alignments of pNA4_D from *C. metallidurans* NA4 and pACP3.3 and pACP4.4 from *A. carolinensis* P3 and P4, pAOVO01 from *Acidovorax* sp. JS42, pALIDE201 from *A. denitrificans* K601, and an unnamed plasmid from *P. pnomenusa* MCB032; Figure S3: Alignment of 221 bp direct repeat flanking large deletion in pNA4_D; Figure S4: Motility assay in soft (0.3%) agar. Diameter measurement of the haloes formed by *C. metallidurans* NA4 and NA4Pt after 24 h; Figure S5: Flow cytometry histogram of *C. metallidurans* NA4 and NA4Pt based on forward-scattered light (FSC) and side-scattered light (SSC); Table S1: List of genomic changes in the laboratory-evolved *C. metallidurans* NA4Pt; Table S2: RNA-Seq-derived up-regulated genes (log2 fold > 0.8; *p* < 0.05) in *C. metallidurans* NA4Pt, compared to its parental strain in non-selective conditions; Table S3: RNA-Seq-derived down-regulated genes (log2 fold < −0.8; *p* < 0.05) in *C. metallidurans* NA4Pt, compared to its parental strain in non-selective conditions.
