# Supplementary material for: Genomic and Transcriptomic Changes That Mediate Increased Platinum Resistance in Cupriavidus metallidurans
Source: Genes (Basel). 2019 Jan 18;10(1):63. doi: 10.3390/genes10010063 (PMC6357080; doi:10.3390/genes10010063)
Supplement: Supplementary file 1 [file genes-10-00063-s001.zip › Figure S3.pdf]

```
52534    GTAGGTATGGCGTCTCCCCTACGGGGCCGGGCTTTCGTGCTGCGCATCGAGCCGCCTGCG    52593
72139    GTCGTTAGGGCGTCTCCCCTGCGGGGCCGGGCTTTCGTGCTGCGCATCGAGCCGCCTGCG    72198
         ** * ** ************ ***************************************

52594    GCGTCTCGCCCCTTCGGGCGTCAATCCCTGACGCCTCCGCGCCGTGCCGGCGCTGCGCGC    52653
72199    GCGTCTCGCCCCTTCGGGCGTCAATCCCTGACGCCTCCGCGCCGTGCCGGCGCTGCGCGC    72258
         ************************************************************

52654    TCCGCTTGCCCGGCCTGAAGGCCGCATCGAGGAACTTTCGCGCGAAAGTTCAGGCTGT      52713
72259    TCCGCTTGCCCGGCCTGAAGGCCGCATCGAGGAACTTTCGCGCGAAAGTTCAGGCTGT      72318
         ************************************************************

52714    GGATCTGATCCGTGGTGTGCCGGCGCTGGTG    52744
72319    GGATCTGATCCGTGGTGTGCCGGCGCTGGTG    72349
         *******************************
```
