# Supplementary figures and images for: Genomic and Transcriptomic Changes That Mediate Increased Platinum Resistance in Cupriavidus metallidurans
Source: Genes (Basel). 2019 Jan 18;10(1):63. doi: 10.3390/genes10010063 (PMC6357080; doi:10.3390/genes10010063)

■ *sil* cluster (ABCD)
 ■ *cop* cluster (WEHQLOFGJIDCBARSNKMTV)
 ■ *ncc/nre* cluster (*nccCBA nreB*)

1

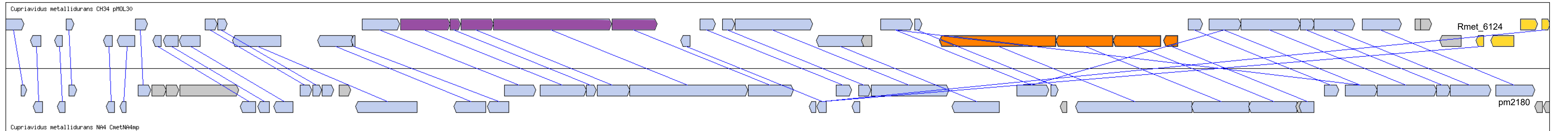

2

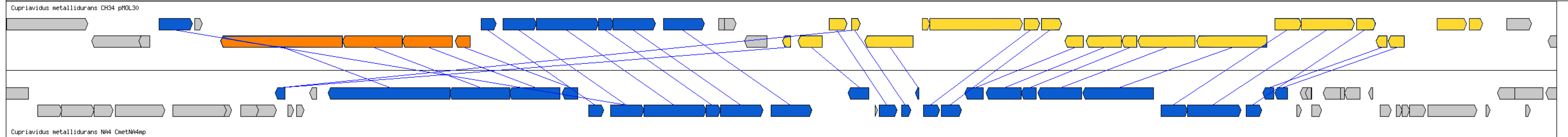

3

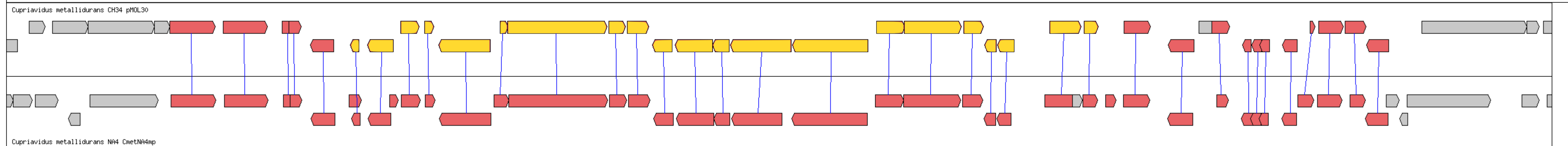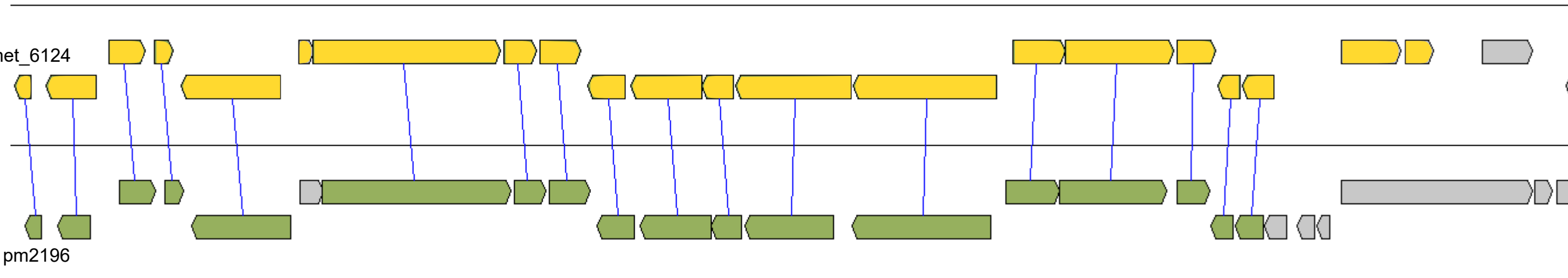

Supplement: Supplementary file 1 [file genes-10-00063-s001.zip › Figure S1.pdf]

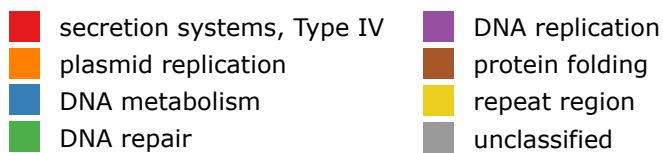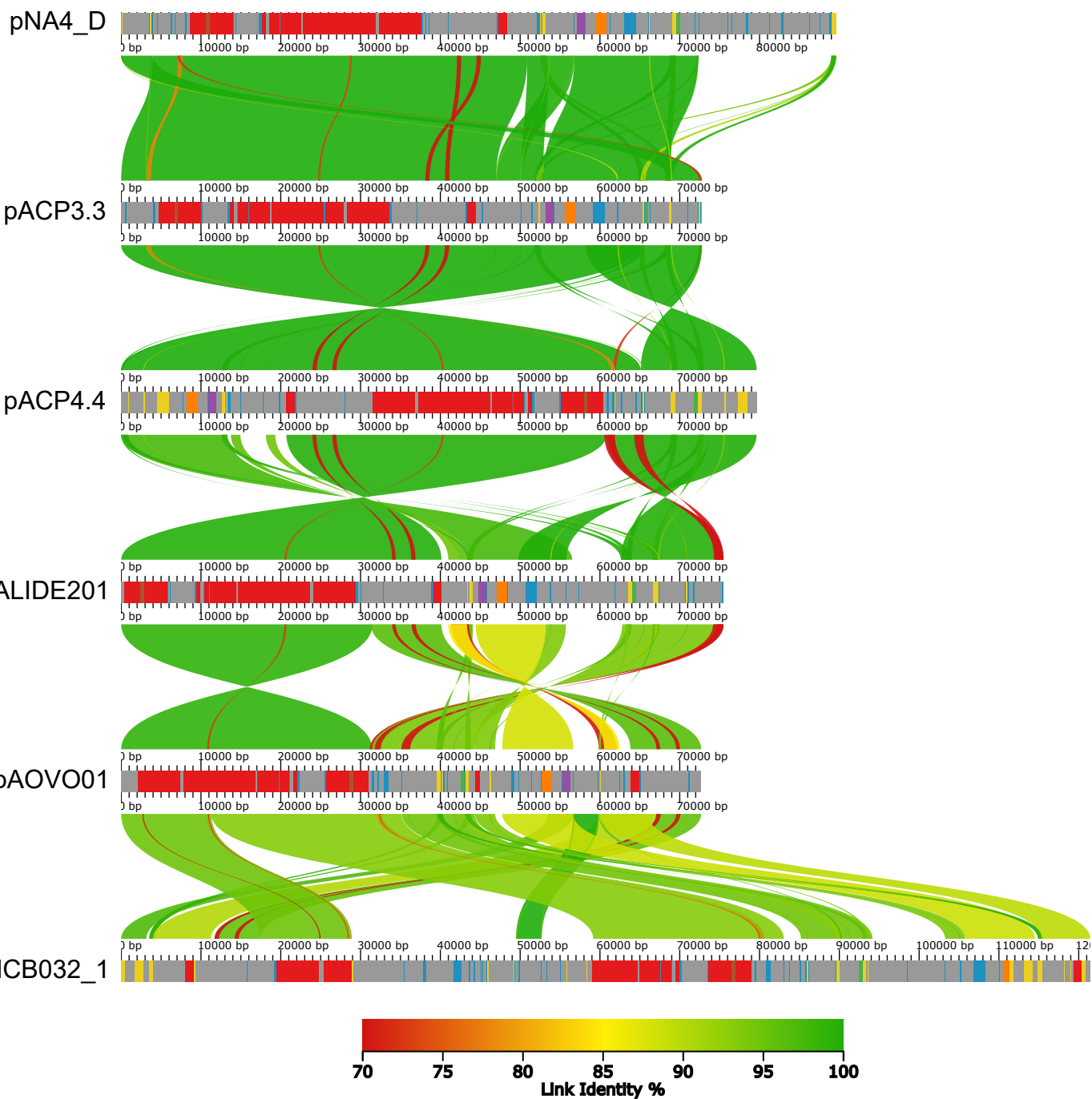

Supplement: Supplementary file 1 [file genes-10-00063-s001.zip › Figure S2.pdf]

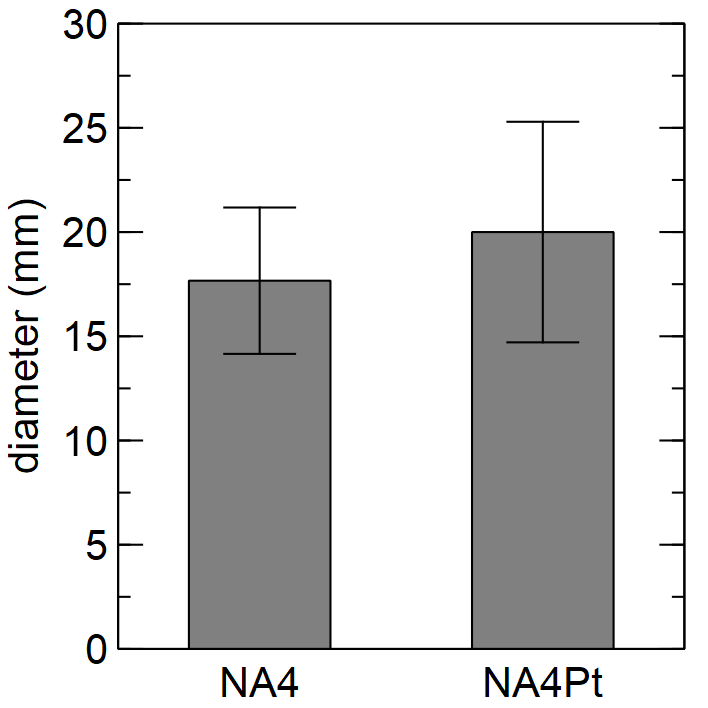

Supplement: Supplementary file 1 [file genes-10-00063-s001.zip › Figure S4.tiff]

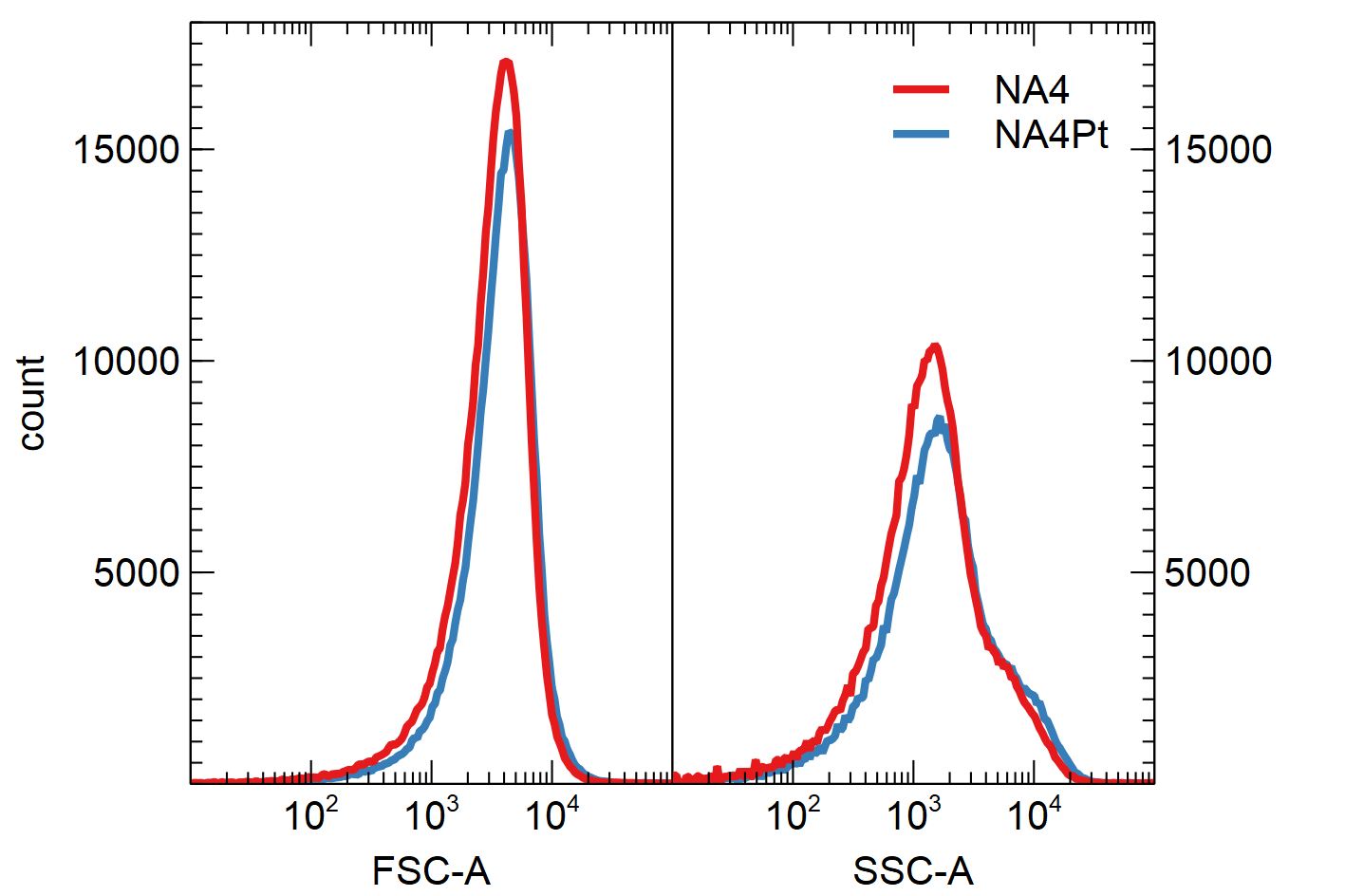

Supplement: Supplementary file 1 [file genes-10-00063-s001.zip › Figure S5.tiff]
